# Neurological Wilson’s Disease Signs—Hepatic Encephalopathy or Copper Toxicosis?

**DOI:** 10.3390/diagnostics13050893

**Published:** 2023-02-27

**Authors:** Anna Jopowicz, Beata Tarnacka

**Affiliations:** 1 Department of Rehabilitation, Eleonora Reicher National Institute of Geriatrics, Rheumatology and Rehabilitation, Spartańska 1, 02-637 Warsaw, Poland; 2 Department of Rehabilitation Medicine, Faculty of Medicine, Warsaw Medical University, Spartańska 1, 02-637 Warsaw, Poland

**Keywords:** Wilson’s disease, hepatic encephalopathy, ATP7B

## Abstract

Wilson’s disease (WD) is a rare autosomal recessive (AR) disorder resulting from mutations in the ATP7B gene, which is responsible for the encryption of transmembrane copper transporting ATPase. The symptomatic presentation of the disease is estimated to be about 1 in 30,000. The impairment of ATP7B function results in a copper overload in hepatocytes, which further leads to liver pathology. This copper overload also occurs in other organs, most particularly in the brain. This could then cause the occurrence of neurological and psychiatric disorders. Symptoms differ substantially and most often occur between the ages of 5 and 35 years. Early symptoms are commonly hepatic, neurological, or psychiatric. While disease presentation is most often asymptomatic, it could also range as far as to include fulminant hepatic failure, ataxia, and cognitive disorders. Various treatments are available for Wilson’s disease, including chelation therapy and zinc salts, which can reverse copper overload through different mechanisms. In select cases, liver transplantation is recommended. New medications, such as tetrathiomolybdate salts, are currently being investigated in clinical trials. With prompt diagnosis and treatment, prognosis is favorable; however, diagnosing patients before the onset of severe symptoms is a significant concern. Early screening for WD could help in diagnosing patients earlier and improving treatment outcomes.

## 1. Introduction

Wilson’s disease (WD) is a rare autosomal recessive (AR) disorder resulting from mutations in the ATP7B gene, which is responsible for the encryption of transmembrane copper transporting ATPase. WD presents mainly with hepatic and neurological symptoms. A large proportion of asymptomatic patients are diagnosed either accidentally, thanks to an elevation in transaminase levels, or during the course of family screening. Hepatic involvement mainly presents with elevated serum transaminase levels alongside related symptoms such as hepatomegaly, portal hypertension, esophageal varices, splenomegaly, ascites, or low platelet level. The other half of symptomatic patients exhibit neurological symptoms such as tremors, dystonia, chorea to drooling, dysphagia, or dysarthria [1,2,3,4,5].

## 2. Hepatic Presentation

Liver disorders are the first symptoms of WD in up to 60% of cases [6]. This form of WD precedes the occurrence of neurological symptoms by almost 10 years [7].

Liver disease in WD encompasses a wide range, from the asymptomatic, who exhibit slight changes in analytical testing such as hepatomegaly or slightly elevated transaminases, to patients with fulminant liver failure. Patients presenting with acute symptoms exhibit similar symptoms to those in acute viral hepatitis such as jaundice and abdominal pain. Symptoms of acute liver injury and acute liver failure, such as coagulopathy and hepatic encephalopathy, tend to occur more often in female patients. Hemoglobin and cholinesterase elevation, and low levels of alkaline phosphatase are typical of WD. Occasionally, hemolytic anemia with a negative Coombs test also occurs, which is regarded as one of the diagnostic criteria of WD [5].

There are very few noted cases of survival in patients with Wilsonian acute liver failure who were unable to receive a liver transplant. Plasmapheresis/plasma exchange has thus far been used with promising results to bridge the gap before transplant [8].

Chronic hepatitis most often manifests initially as a slight transaminase elevation that then steadily progresses to fibrosis and cirrhosis. The appearance of cirrhosis causes an increased risk of mortality [9,10]. The differential diagnosis of liver presentation should include viral hepatitis, nonalcoholic fatty liver disease (NAFLD), autoimmune hepatitis (AIH), and metabolic liver diseases [5,11].

## 3. Hepatic Encephalopathy

Hepatic encephalopathy (HE) is neurological dysfunction resulting from liver insufficiency and/or portosystemic shunting. Manifestations range over a wide spectrum of neurological/psychiatric abnormalities [12,13]. The mildest forms of HE only slightly impair some cognitive abilities, which can only be detected via the use of extremely sensitive psychometric neuropsychological tests. These tests assess the complex subsystems of attention, working memory, psychomotor speed, some forms of visuospatial ability, and electrophysiological and other functional brain measures. With regard to the sleep–wake cycle, daytime somnolence frequently occurs, whereas the reversal of the sleep–wake cycle is less often seen [14].

Noncomatose patients with HE could present with motor-system disorders such as hypertonia, hyperreflexia, and a positive Babinski sign, and the absence of deep tendon reflexes could be noted. On rare occasions, transient focal neurological deficits have also been exhibited [15]. Other rare symptoms include involuntary movements such as tics or chorea. Occurring more commonly are symptoms of extrapyramidal dysfunction, for example, hypomimia, muscular rigidity, Parkinsonian-like tremors, monotonous speech, and hypo-, brady-, and dyskinesia with reduced voluntary motions.

The ’flapping tremor’ that often occurs in early stages of HE is a negative myoclonus involving the loss of postural tone and not a tremor [11,14,15,16]. 

The most common classification system used in grading HE is the West Haven criteria. According to this system, there are four grades of the clinical manifestation of HE. Grade I involves subtle personality changes that are mainly only noticed by the patient’s family members, and a shortened attention span. In Grade II, time disorientation occurs alongside inappropriate behavior, lethargy, or apathy. Grade III patients are additionally space-disoriented, exhibit bizarre behavior, and are semistuporous but still responsive to stimuli. Grade IV patients are comatose and do not react to pain stimuli. Grade V, subclinical or minimal HE, was added recently to include patients who had altered brain function in neuropsychological/neurophyssssiological tests without exhibiting clinical signs of HE [17,18,19]. HE diagnosis is based on four factors: a characteristic clinical pattern, the presence of severe liver failure and/or portal systemic shunt alongside high ammonia levels, no alternative causes for the aforementioned disorders, and the patient’s response to ammonia-lowering treatments. Upon the confirmation of the diagnosis, five more factors need to be taken into consideration: underlying conditions, the severity of mental disruptions, the timeline of the symptoms, precipitating events, and the response to treatments [12].

As the clinical symptoms and findings observed in patients with HE are not specific, a diagnosis in each individual case can only be conducted upon the exclusion of other possible causes of brain dysfunction. Useful diagnostic tools include magnetic resonance imaging (MRI), single-photon emission computerized tomography (SPECT), and cerebrospinal fluid (CSF) analysis. However, the response to treatment may prove to be the best way to confirm the diagnosis [19,20] Cognitive dysfunction that often precedes other symptoms of HE may be detectable only via the use of psychometric tests [19].

Possible symptoms and precipitating factors to be considered during the diagnosis of HE include intracranial bleeds, gastrointestinal (GI) bleeding, infection, and electrolyte imbalance. The presence of a focal neurological deficit means that an alternative diagnosis must be taken into consideration [21].

Neuropsychological tests such as the seven-pointed star, serial 7 s, and the animal naming test [22] can be employed to detect neurocognitive dysfunction in cirrhotic patients with suspected low-grade HE. Number-connection tests A and B (trails test) are two of the tests used in evaluating neurocognitive impairment in these patients. Neuropsychological tests are mainly employed in diagnosing covert HE. Electroencephalography (EEG) can be used to detect changes in the cortical cerebral activity of patients with HE. The test, while not reliant on patient cooperation, can be influenced by metabolic disorders and the use of medications. EEG is also relatively insensitive for patients with covert or low-grade HE [23].

## 4. Neurological Manifestation of Wilson’s Disease

The first neurological symptoms of WD most often occur between the ages of 20 and 30 years. Some of these symptoms are tremors, Parkinsonian-like symptoms such as hypomimia and drooling, ataxia, and dystonia [24,25].

In 22–55% of cases of neurological WD, patients present with tremors in one or both hands. Several tremor subtypes such as rest, action, and Holmes tremors may occur in patients with neurological WD. Resting tremors, typical of Parkinson’s disease, are less common and, when present, are normally linked with postural and action tremors of greater severity.

A characteristic tremor in WD is the “wing-beating” tremor, best induced when the arms are held forward and flexed horizontally, bringing out a proximal tremor that resembles the beating of a bird’s wings. When such a tremor is seen, imaging studies may reveal a lesion in the dentatorubrothalamic pathway. However, this tremor occurs less frequently than other tremor subtypes do in WD.

Akinetic–rigid forms of parkinsonism with bradykinesia, rigidity and gait difficulty occur in 19–62% of WD patients. Ataxia occurs as a variable symptom of WD. Limb ataxia is rare, but may occur alongside dysmetria of the eyes and ataxic dysarthria [26,27].

Dystonia is the most severe neurologic presentation of WD. It is found in 10–30% of patients and presents in different forms: focal, segmental, or generalized [13].

Focal dystonia (including facial dystonia, risus sardonicus, orofacial dystonia, limb dystonia, dystonia of the vocal cords, dystonia of the pharyngeal muscles, blepharospasm, cervical dystonia, and writer’s cramp) may be unilateral at disease onset, but can further progress to a bilateral or even generalized involvement. Severity ranges from mild to severely debilitating, such as the occurrence of opisthotonus. Status dystonicus involving frequent and relentless episodes of devastating generalized dystonia may also occur [27,28]. Chorea and athetosis occur in about 10% of cases with WD. The choreoathetoid form of WD is more common in patients with young onset disease, occurring in about 20% [27].

Dysarthria in WD patients can manifest as mixed dysarthria with varying degrees of spastic, ataxic, and dystonic features.

Ophthalmological manifestations of WD include KF rings and sunflower cataracts. KF rings represent copper deposition in the corneal Descemet’s membrane, and they can be visible to the naked eye as greenish or brownish discoloration at the outer circumference of the cornea. The rings do not impair vision and clear with chelation therapy. Sunflower cataracts also do not impair vision. However, slit-lamp examination is essential for detection. Extraocular movement abnormalities rarely occur and, when present, are secondary to copper deposition in the midbrain. The absence of nystagmus is useful in diagnosing WD [27].

There are a number of classification systems used in grading the severity of neurological impairment. The Unified *Wilson’s Disease* Rating Scale (UWDRS) takes three factors into consideration: (1) consciousness, (2) changes in daily life activities, and (3) the results of clinical neurological examination [29].

Another scale is the Global Assessment *Scale* for *Wilson’s* Disease (*GAS* for WD), which assesses global disability (Tier 1: liver, cognition, behavior, motor function, and osseomuscular) and the nervous system (Tier 2: neuropsychiatric).

Neither of these systems include assessment of liver function tests and as such cannot be used in the differentiating HE from neuropsychiatric WD [25,30].

## 5. Psychiatric Manifestations in Wilson’s Disease

Psychiatric symptoms are common in adult patients with WD. They are present in 25% of patients at initial diagnosis. In later stages of the disease, they occur in almost 100% of adult patients.

The main symptoms are the following:

(1) Behavioral disturbances.
(2) Mood disturbances (including mania, depression).
(3) Cognitive deficits (involving mainly executive and visuospatial function disturbances).
(4) Other symptoms such as psychosis and sleep disturbances [5].

Behavioral and personality disorders occur in 46–71% of WD patients, and the most common manifestations include irritability, aggression, and antisocial behavior. Behavioral and personality disturbances potentially lead to severe social problems, such as family difficulties (divorces, unemployment, or even criminal activity) and difficulties in receiving medical care, including treatment, diagnosis, and rehabilitation (issues include verbal aggression and noncompliance with anticopper treatment and other medical recommendations) [31].

Mood disturbances are the most common psychiatric manifestation in WD. Of WD patients, 20–60% develop depression over the course of the disease. A high rate of suicide attempts, ranging between 4% and 16% of WD patients, was also reported [32,33,34].

Bipolar disorder also occurs more frequently in WD (14–18%) than in the healthy age- and sex-matched population [35].

Mood disorders, especially depression, are often undiagnosed in patients with chronic neurological illnesses. The use of scales such as the Hamilton Depression Scale or Montgomery–Asperg Depression Rating Scale to monitor symptom severity could be clinically valuable [31]. Depression was often described in the beginning of the illness and could thereby not be attributed to the psychosocial consequences of a chronic medical disease. Often, patients with psychotic symptoms are started on antipsychotic medications and subsequently develop significant neurologic symptoms that are initially interpreted as side effects related to antipsychotics. When these side effects present unusually, neurologic consultation is requested, and this sometimes leads to the diagnosis of WD [36].

Current epidemiological studies suggest that psychosis in WD occurs rarely and is not more frequent than that in the general population; however, it occurs more frequently in patients with a neurological WD manifestation. Clinical manifestations of psychosis in WD are nonspecific, and patients’ diagnoses include schizophrenia, and schizoaffective and/or delusional disorders. Psychotic symptoms occurring as the first manifestation of WD could present both diagnostic and therapeutic challenges [31].

Cognitive disorders occur in about 25% of patients [37], and include declining school performance in children and executive function impairment in adults [13]. The affected cognitive domains are responsible for attention, visuospatial perception and reasoning, learning and memory, and verbal and abstract reasoning. Most often, these cognitive deficits are mild and potentially reversible at disease onset, but can also further deteriorate during the disease, leading to some patients eventually being diagnosed with mild cognitive disorder or even dementia. In contrast to neurodegenerative dementias, intellectual deficits presenting in WD patients are a result of metabolic changes, similarly to those occurring in hepatic encephalopathy or manganism, and are potentially reversible with anticopper treatment [38,39,40].

WD patients may exhibit slow processing speed and mild impairment in all cognitive domains, particularly in working memory, attention, and abstract thinking. These cognitive deficits are most likely due to lesions in the corticostriatal pathways. They also correlate with the global severity of MRI abnormalities such as hyperintense lesions, basal ganglia metal deposition, and subcortical and cortical atrophy. The direct effect of toxic copper is known in the etiology of cognitive deficits in WD. The pathological effect of toxic free copper on neurons and cognitive function was also demonstrated in AD, and higher levels of free copper were correlated with a negative evolution of cognitive deficits. The presence of subclinical hepatic encephalopathy may be regarded as an additional risk factor for cognitive dysfunction development [31,41]. WD patients (when not in advanced stages of HE) usually do not present with severe consciousness disorders [42]. Proper psychological measurements should include the assessment of various types of attention (vigilance, sustained, selective, and divided attention), memory and learning, perception, praxis, construction, information processing speed, working memory, verbal fluency, spatial ability, cognitive flexibility, problem solving, and other executive functions. Emotional–behavioral functioning should also be assessed during cognitive testing [13].

Sleep disturbances such as poor quality of sleep, frequent nocturnal awakening, and other sleep disturbances such as sleep paralysis were described in WD patients [43,44].

Insomnia, daytime sleepiness, cataplexy-like episodes, restless leg syndrome (*RLS*) and REM sleep behavior disorder (*RBD*) occur more frequently in WD patients than they do in the general healthy population. Sleep disruption most likely stems from copper deposition and consequent neurodegeneration involving the brainstem. Poor nocturnal sleep quality with consequent higher daytime napping in WD could be the result of motor symptoms, as dystonia and tremors interfere with falling asleep. RLS is probably related to the high iron concentrations and neuronal loss in the substantia nigra, and to the dopaminergic dysfunction detected at pre- and postsynaptic levels in the basal ganglia [41,45,46].

Some other psychiatric conditions including catatonia, anorexia nervosa, bulimia, obsessive–compulsive disorder and attention deficit hyperactivity disorder (ADHD) have also been reported in WD.

In summary, taking into consideration the wide spectrum of psychiatric symptoms observed in WD patients, WD should be included in the differential diagnosis of young adults presenting with the first episode of psychiatric symptoms, and should be suspected in all young adult psychiatric patients that additionally manifest extrapyramidal and/or hepatic symptoms [31,47,48].

## 6. Diagnosis

Diagnosis is based on clinical presentation and brain imaging [6]. However, as mentioned earlier, it can be very difficult to classify the patient’s neurological symptoms due to mixed presentations [25].

WD is diagnosed on the basis of diagnostic algorithms that consider clinical symptoms, measures of copper metabolism, and DNA analysis [1].

Serum ceruloplasmin levels are typically lower in patients with neurologic WD, but they may be within the low normal range in about half of those with active liver disease [49]. Enzymatic or nephelometric immunoassays can be used to measure serum ceruloplasmin when WD is suspected [50].

Total serum copper levels are usually decreased in proportion to reduced ceruloplasmin in the circulation. However, normal or elevated serum copper levels with reduced ceruloplasmin levels indicate an increase in the concentration of copper that is not bound to ceruloplasmin in the blood (non-ceruloplasmin-bound copper), which is suggestive of WD. Non-ceruloplasmin-bound copper (NCC) concentration can be estimated by subtracting ceruloplasmin-bound copper from the total serum copper concentration [51].

A 24-h urinary copper excretion test can help in diagnosing and monitoring treatment in WD. The conventional level taken as the diagnostic of WD is >100 μg/24 h (>1.6 μmol/24 h) in symptomatic patients [1]. However, the basal 24-h urinary copper excretion may be a fivefold increase in urinary copper excretion after an oral d-penicillamine challenge has been used for diagnostic purposes in children [52]. Although this test is used in some centers, its value has not been confirmed, and it may not be required if the lower threshold for urinary copper excretion of 40 μg/24 h is applied [53,54].

Mutational analysis is another important diagnostic tool. However, the results of direct molecular–genetic diagnosis may take time to obtain; analysis is challenging because there are over 700 possible mutations, and many patients are compound heterozygotes. Sequencing is increasingly faster and cheaper, and will become a commonly used diagnostic test [55,56].

Typically, WD patients with neurological symptoms present with symmetrical T2 hyperintensity or mixed intensity in the putamina (with hyperintense peripheral putamina rim), globi pallidi, caudate nuclei, thalami, and pons in MRI testing. The midbrain, cerebellum, corticospinal tracts, cortex, and subcortical area are also affected. WD patients with neurological symptoms exhibit brain MR abnormalities in about 100% cases, patients with hepatic symptoms in 50%, and resymptomatic patients in about 20% of cases.

Liver histology can reveal various abnormalities such as inflammation, portal fibrosis, microvesicular and macrovesicular steatosis, or cirrhosis and should not be used to confirm diagnosis. Neurological patients usually present with liver damage upon histological examination [5]. Biochemical tests including aspartate transaminase, alanine aminotransferase (ALT), alkaline phosphatase (ALP), gamma glutamyl transferase (GGT), serum bilirubin with conjugated fraction, serum albumin, prothrombin time and glucose should be carried out to assess hepatic synthetic function and the extent of liver. The acute presentation of WD with liver failure presents high total bilirubin levels (>300 mcmol/L, >17.5 mg/dL), relatively low serum transaminase levels (100–500 IU/L), and low serum alkaline phosphatase level, resulting in a low alkaline phosphatase (IU/l)-to-total bilirubin (mg/dl) ratio (<1). Coombs negative hemolytic anemia may be the first manifestation. Thrombocytopenia is associated with hypersplenism resulting from established portal hypertension [51,57,58]. The association of three key biochemical markers (low serum caeruloplasmin, low serum copper, and high urinary copper excretion) is highly predictive of WD diagnosis. Caeruloplasmin levels may be normal or elevated due to hepatic or other inflammatory conditions, and low as a result of malnutrition and, in 15% of carriers, due to WD mutations [5,6] The use of the radioactive copper test in WD diagnosis is also worth discussing. Historically, before the discovery of the ATP7B gene, the incorporation of radioactive copper (Cu64 or Cu67) into ceruloplasmin was used in WD diagnosis. It is currently used in a few centers, and its sensitivity and specificity for WD diagnosis are 98.6% and 100%, respectively. In selected cases where the final diagnosis of WD is difficult to establish, performing the test should be considered [5,59,60].

Molecular testing for ATP7B mutations is essential and, in most cases, confirms the diagnosis of WD. Direct DNA sequencing can improve mutation detection (up to 95%), but carries the risk of identifying variants of unknown significance, thus leading to additional diagnostic difficulties. The identification of one disease-causing mutation confirms the diagnosis of WD only if specific clinical symptoms and biochemical signs of an impaired copper metabolism are also present [6,61].

## 7. Differential Diagnosis of Hepatic Encephalopathy and Wilson Disease

The differential diagnosis of hepatic encephalopathy and Wilson disease includes:

- Clinical symptoms.
- Laboratory test.
- MRI.
- Proton magnetic resonance spectroscopy.

### 7.1. Clinical Symptoms

HE and WD have several clinical neuropsychiatric signs and symptoms in common during the milder stages of the disease. As such, the differentiation of these two disorders based on clinical symptoms is often impossible [13].

The differential diagnosis of WD with neurological presentation includes movement disorders, especially: Parkinsonian and Parkinsonian-plus syndromes, neurodegeneration with brain iron accumulation, inborn dystonia (DYT), and multiple sclerosis. The Kayser–Fleischer (K–F) ring and sunflower cataracts are pathognomonic for WD, but they very rarely occur in children. K–F rings occur in almost 100% of nontreated patients with neurological presentations of WD, in 50% of patients with hepatic presentation, and even in 20% of presymptomatic patients [5].

### 7.2. Laboratory Test

In untreated WD patients, the 24-h urinary excretion of copper is elevated and corresponds to the NCC fraction in the serum [62]. Urinary copper, therefore, plays an established role in diagnosis. A comprehensive 24-h collection test that includes the exact volume and total creatinine excretion/24 h is required to obtain accurate results. Additionally, the copper contamination of the urine collection device should be avoided. Typical ranges for the 24-h urinary collection for copper in WD patients vary among laboratories, but are generally about >1.6 µmol/24 h (>100 µg/24 h). However, other liver diseases can cause false-positive 24-h urine copper levels. In autoimmune hepatitis, cholestasis, and chronic or acute liver failure, urinary copper excretion can be increased without WD [49,63,64]. Increased urinary copper excretion may be a compensatory response to impaired biliary excretion of copper in these cases. Unlike serum NCC, the urinary copper excretion in treated patients is also dependent on chelating agents, which limits its role in treatment monitoring [65].

### 7.3. MRI

MRI changes can be present in WD even before neurological or liver manifestations are detected and some may be reversible following copper chelating treatment. High-signal-intensity lesions in the basal ganglia on T1-weighted images generally are secondary to chronic liver disease. High-signal-intensity lesions on T2-weighted images reflect yjr cerebral involvement of WD and typically include changes in deep gray matter (putamen, caudate and thalamic nuclei), and mesencephalic and pontine white matter, and atrophy. These changes have not been reported in children with WD [6,66]. In WD, abnormal bilateral T1 and T2 signals, especially in the basal ganglia (putamen), with or without brain stem impairment, have become one of the important signs of WD [67]. Aberrant signals in the basal ganglia can be the effect of glial cell hyperplasia, edema, and necrosis caused by copper depositions with the possibility of disappearing (edema and demyelination) after proper decoppering therapy [1].

Patients with mild HE do not have evident cognitive impairment, and it is only detectable via some psychometric tests such as the critical flicker frequency test or psychometric hepatic encephalopathy score. It is still difficult to distinguish patients with mild HE from those without HE. There is no gold standard in the diagnostic criteria of HE. It is also very important to diagnose mild HE symptoms because patients with mild HE may develop overt HE. It is, therefore, very important to diagnose the early stage of HE to avoid future cognitive complications.

### 7.4. Proton Magnetic Resonance Spectroscopy

*Proton magnetic resonance spectroscopy* (proton MRS) (1 H MRS) has been a noninvasive method of measuring brain metabolites since the late 1980s. Spectroscopy improves clinical diagnosis, and can be used in treatment effect monitoring and understanding disease mechanisms. MRS is a noninvasive method for the evaluation of HE treatment strategies, especially if using high-field magnets. MRS enables the quantitative assessment of different peaks. Proton MRS in the brain mainly detects lactate (an end product of anaerobic glycolysis), N-acetyl aspartate (NAA, a neuronal marker), myo-inositol (putative glial marker with unknown function), glutamate and glutamine (substrate and precursor of the amino acid involved in the excitatory neurotransmission), creatine and phosphocreatine (metabolites of energetic reservoir), and choline derivates (cell membrane components) [68].

MRS followed the alterations of brain metabolites in patients with cirrhosis. Proton spectroscopy studies showed a progressive increase in glutamine and glutamate peaks according to the severity of HE [69]. The clinical resolution of an episode of HE was associated with a decrease in glutamine and glutamate peaks after treatment [69]. Myo-inositol also had a tendency to gradually decrease with HE degree, even though there were no changes in overt HEs. These changes are associated with compensation in the brain due to the regulation of cerebral osmolytes produced by an increase in ammonia. Studies did not demonstrate any changes in either choline or NAA except in advanced stages of HE [70]. Hermann et al. observed, in patients with mild HE, T1-weighted hyperintense signals in MRI in both pallidum in 18 (58%) on MRI, but MRS showed an HE profile in 19 (61%). Of MHE patients, 19 (95%) had typical MRS HE abnormalities according to the radiological interpretation. In that study, the spectroscopic profile showed increased levels of glutamine/creatine and glutamate/creatine, and decreased myoinositol/creatine levels; choline/creatine levels did not significantly change. The authors stated that MRS could be more sensitive in the detection of mild HE brain changes [71].

In patients with WD, contrary to patients that presented only with HE, an NAA decrease was seen in all patients with hepatic and neurological presentation, with statistically lower values of mI/Cr and NAA/Cr within the globus pallidus profile, and higher Lip/Cr in relation to the control group [72]. These results suggest the loss of neurons in all WD groups, contrary to patients with HE, in whom only in severe neurocognitive impairment was such a pattern detected. The severity of the neurological status and the severity of liver damage were correlated with the severity of changes in 1H-MRS in WD [72]. Within the globus pallidus, a decrease in NAA/Cr concentration was corelated to the severity of the clinical symptoms in the neurological and hepatic WD subtypes (r = −0.6563 and r = −0.9827, respectively; *p* < 0.01) [73]. In patients with WD with liver symptoms after one year of treatment, MRS showed a significant increase in mI/Cr and Glx/Cr, while patients with neurological symptoms had an increase in NAA/Cr [73]. This indicates the resolution of osmolyte regulation and metabolic changes within the mitochondria of neurons. Our study also suggests that, in WD, MRS may present greater values in trying to distinguish HE-related lesions from lesions associated with neuronal damage. We hypothesize that, in patients with neurological symptoms, there would be impaired astrocytic–neuronal cooperation as a result of liver damage (decrease in NAA/Cr and increase in Glx/Cr as a result of increased glutamine) and the damaging effect of free copper ions (membrane breakdown—increase in Cho/Cr). The decrease in NAA/Cr in these patients correlated with the severity of neurological status, but was reversible, as mentioned before. Therefore, as a result of the damage of astrocytic cells in early WD stages with the predominance of HE, there would be a subsequent reversible disturbance of neuronal metabolism in WD patients. In more severe stages resulting from copper deposits, further neuronal destruction can occur, but with permanent neuronal damage.

Voxel-based morphometry is a new MR strategy to perform volumetric studies of the brain and subsequently address brain atrophy. MR experiments with this approach showed that cirrhotic patients had many areas with a decrease in gray matter volume and an increase in white matter volume in comparison to those of healthy controls, which were further aggravated with HE progression [74]. Tarasow at al. showed that there were no differences in atrophy between cirrhotic patients and those with overt HE [75].

## 8. Treatment

Conventional drug therapy for WD involves the removal of copper excess (decoppering) via the promotion of copper excretion with the use of chelating agents such as D-penicillamine and trientine, via blocking intestinal copper absorption with zinc salts, or the concurrent use of both methods. Penicillamine is used as first-line treatment in acute and/or symptomatic WD [76,77]. It binds to copper and is excreted via urine. While highly effective, serious adverse side effects have led to the discontinuation of the drug in up to 30% of patients. The paradoxical worsening of the neurological symptoms of WD following the introduction of penicillamine therapy has been well-described in the literature, and improves or stabilizes upon the discontinuation of the drug. Patients with chronic liver disease, leucopenia, and/or thrombocytopenia carry the highest risk of developing severely adverse drug reactions. Trientine dihydrochloride is used as a second-line drug for patients with penicillamine intolerance. It promotes copper excretion via the kidneys and is equally effective, and its side effects, while similar to those occurring during penicillamine use, manifest less frequently. Trientine tetrahydrochloride (Cuprior) is a hybrid medicine containing a different form of trientine with similar benefits and risks to those of trientine dihydrochloride. The main advantage is improved trientine release in the body, which means that lower doses are able to stimulate a good patient response. This renders it more cost-effective [53,78].

Zinc salts are used in combination with penicillamine for the initial management of symptomatic patients [79,80,81]. They are effective and safe as a first-choice treatment in presymptomatic patients, and as maintenance therapy for stable patients on penicillamine or trientine. They could be considered first-line therapy in patients with neurological symptoms. The working mechanism involves the activation of metallothionein in intestinal cells, thereby enhancing copper binding in enterocytes and the reduced absorption of copper from the gut [82,83]. Bis-choline tetrathiomolybdate (TTM; Decuprate) is a new drug for WD patients presenting with acute neurological disease because conventional chelator therapy could lead to rapid and irreversible clinical deterioration. Phase III clinical trials are being carried out to assess its efficacy and safety in adult WD patients. The treatment of either liver or brain disease manifestations of WD should be lifelong and uninterrupted. The discontinuation of maintenance treatment results in the recurrence of symptoms, liver failure, and further neurological deterioration [6,84,85].

## 9. Discussion

Wilson’s disease (WD), unlike many other neurogenetic metabolic diseases, can be effectively treated in both the acute and chronic stages of the disease. However, the identification of WD remains challenging due to its nature as a great imitator, which requires a high level of suspicion to achieve a correct and timely diagnosis. In approximately 40–50% of patients, initial neurological problems are observed, while the rest present with either hepatic or primarily psychiatric manifestations [86]. Neurological and neuropsychiatric problems associated with WD are nonspecific, and many patients with neurological symptoms do not exhibit obvious hepatic symptoms. The most common neurological abnormalities include dysarthria, dystonia, tremors, and parkinsonism [86]. The diagnosis of WD is often fraught with the risk of misidentification due to the varied nature of the symptoms. As such, laboratory tests indicative of abnormal copper tissue levels are the basis of WD diagnosis. These tests include ceruloplasmin measurement, the measurement of urinary copper excretion, copper quantification in liver biopsies, and genetic assessment. Useful diagnostic tools include MRI and SPECT. The diagnosis is then performed on the basis of a combination of the results of the aforementioned tests and clinical symptoms. Proton magnetic resonance spectroscopy (1H-MRS) is a noninvasive resonance imaging method that analyzes the chemical composition of tissue and could be able to detect the metabolic abnormalities of neural tissue in WD in a timely manner, since changes in tissue metabolism often precede the structural changes seen on standard magnetic resonance imaging (MRI). Data from the literature show that 1H-MRS of the brain is useful in predicting the progression of many neurological diseases and monitoring treatment. The 1H-MRS is also sensitive in the detection of HE. It could be helpful in differentiating changes in the brain associated either with the presence of HE or the toxic effects of copper alone in patients with WD [10,86].

Mutations in the ATP7B gene and the subsequent deactivation of the ATP7B transporter lead to impaired biliary copper excretion. As ATP7B also oversees the transportation of copper during the synthesis of ceruloplasmin, mutations in the gene lead to decreased ceruloplasmin formation. Nonetheless, toxic ceruloplasmin-bound copper levels are commonly elevated [1].

Effectual treatment options are available and, if introduced sufficiently early, are able to mitigate the myriad of hepatic and neuropsychiatric presentations of the disease [87].

Pharmacological means of therapy include the use of chelating drugs such as D-penicillamine or trientine and zinc salts. Liver transplantation is also a treatment option for cases involving end-stage or acute liver failure [10,88].

New drugs such as tetrathiomolybdate salts are currently being tested in clinical trials alongside genetic therapies in animal models. The importance of early treatment, before the onset of severe symptoms, as a means of halting disease progression cannot be overstated. As such, advances in WD screening could feasibly improve disease diagnosis and management [1,89].

The pharmacological means of treatment are based on ensuring negative copper level balance without the induction of iatrogenic copper deficiency. These treatment methods are essentially lifelong, and the most effective method is excess copper chelation. However, certain chelators may cause additional clinical deterioration in some patients [86].

## 10. Conclusions

While WD is progressive with a fatal outcome when left untreated, proper treatment and diagnosis ensure the successful management of its symptoms. Unfortunately, the wide variation in the initial symptoms could delay the right diagnosis of the condition and thereby treatment. The diagnosis of Wilson’s disease (WD) is based on several criteria, including increased urinary copper excretion, reduced serum ceruloplasmin levels, the detection of the Kayser–Fleischer ring in the cornea through slit-lamp examination, and liver biopsy findings. Magnetic resonance imaging (MRI) findings in WD can vary from normal to symmetric areas of T1 hyperintensity affecting the basal ganglia (BG), thalami, external capsules, pons, and midbrain. In some cases, T2-weighted confluent hyperintensities may be found in the subcortical white matter and cortical gray matter of parietal, temporal, and frontal lobes. Unlike conventional MRI, magnetic resonance spectroscopy (MRS) provides information on neuronal/axonal viability, cellular membrane status, and cellular energetics. Therefore, the brain MRS can be a valuable diagnostic tool for the early detection of neurological changes in children with Wilson’s disease [90].

Pharmacological treatments for WD must be lifelong and aimed at restoring negative copper balance without inducing iatrogenic copper deficiency. The chelation of excessive copper is considered the gold standard of therapy, but chelators may induce further clinical deterioration in some treated patients.

## Data Availability

Not applicable.
